# The Hospital World

**Published:** 1911-07-22

**Authors:** 


### The Hospital World.

### Dr. Hermann Adler.

We regret to record the death of the Very Rev. Dr. Hermann Adler, C.V.O., D.C.L., LL.D., who for upwards of twenty years has fulfilled the responsible duties of Chief Rabbi and has deservedly won the appreciation and good will of men of all shades of opinion and of all classes of people. He was a learned man with a kindly genial nature, who was full of good works and always ready to do personal service in furtherance of any cause calculated to help the people. It was remarkable that he should have been able to devote so much time to public work of all kinds, and we are afraid that his desire to help wherever possible tended, especially in these latter years, to deplete his energy to the vanishing point. He was a member of the Council of the Hospital Sunday and King Edward's Hospital Funds, and was never too busy to devote time in the cause of the voluntary hospitals. We offer our sincere sympathy to Mrs. Adler and the family, to whom Dr. Adler's death must have come as a grievous blow. Dr. Adler's life has been full of useful work which will make his memory treasured by a multitude of people representing very varied types of human activity.

### From Aberdeen to Chester.

Dr. David Rennet, assistant deputy medical officer of health for Aberdeen, has been appointed medical officer of health for Chester. Dr. Rennet has been connected with Aberdeen since 1889, when he graduated M.B. and C.M. at its university, and gained the D.P.H. In 1892 he was appointed assistant professor of medical jurisprudence and public health to the university.

### Thirty Years of Municipal Medicine.

Dr. J. W. Mullen, Medical Superintendent of the Ladywell Sanatorium, Salford, has just completed thirty years' work for the Salford Corporation. When the Ladywell Sanatorium was opened Dr. Mullen was appointed Medical Superintendent, but his connection with Salford is older still. Coming from Dublin in the first place, he was appointed in 1881 assistant to Dr. Tatham, the medical officer of the borough. In those days Dr. Mullen's work was carried on at Wilton Hospital in Cross Lane. Nine years later, when Dr. Tatham was appointed Medical Officer to Manchester, Dr. Mullen became Medical Superintendent, a post he continued to hold on the opening of the new institution at Ladywell. Practically all the infectious cases of the district, except smallpox, which is relegated to Drinkwater Park at Agecroft, are treated by arrangement at Ladywell. It is often pointed out as a good example of a municipal hospital. Dr. Mullen, however, has not allowed this work to absorb all his energies, for he has a reputation in the neighbourhood as an amateur of music and acting.

### San Giovanni Hospital, Turin.

The following conclusions have been arrived at by the committee appointed by the corporation to consider the question of hospital maintenance in Turin. For the proper adjustment of the maintenance problem the overcrowding of the hospital of San Giovanni must be done away, as regards the respective classes of convalescents and incurables. Such a reform would give increased power to the hospital to deal with some 2,000 fresh patients in the year. Nevertheless, the prevention of the overcrowding is not to stand in the way of the solution of the other problems concerned in the general maintenance question. For the solution of these a comprehensive scheme is necessary, which shall include the assignation of 800,000 lire (£32,000) out of the hospital maintenance fund to the hospital of San Giovanni, so that the minimum scheme presented to the head of the administration of the hospital in November last may be carried out, and in addition the pressing on the Junta of a scheme for the solution of the remaining problems on the basis approved by the Communal Council in 1904 which dealt with the hospital for chronic cases, the maternity hospital, and the San Lazzaro.

### Baron Merthyr and the Hamadryad Hospital, Cardiff.

Baron Merthyr of Senghenydd, or, to call him by the old title under which he has been known and respected for many years, Sir William Thomas Lewis, besides being one of the great figures in the industrial world of South Wales, has always taken a keen interest in the local charities.

He has acted as President of the Royal Hamadryad Seamen's Hospital in Cardiff, and done everything for its welfare since its establishment in 1866. Previous to that date sick and injured seamen had been treated at their lodging-houses by local practitioners. This system had proved not only very unsatisfactory in itself, but had also entailed highly disproportionate expense. In order to remedy these defects the leading shipowners and merchants of the port determined to establish a seamen's hospital at which sickness and accidents of all kinds could be treated efficiently and economically, and by which the spreading of infectious diseases amongst the inhabitants might be prevented. The Government, on representations made to it, placed H.M.S. *Hamadryad* at their service. The vessel accordingly was brought round from Devonport and equipped as a floating hospital at a cost of £2,800, which was generously defrayed by the Marquess of Bute and some of the merchants and shipowners of the port. It was found necessary in later years, owing to the great increase in the applications for admission and to the age and decay of the ship, to build a permanent Seamen's Hospital on shore. Meetings were held by the Chamber of Commerce, the Shipowners' Association, and also by the townspeople to consider this question, and a resolution was adopted unanimously at each of these meetings to commemorate the sixtieth year of the reign of Queen Victoria, the longest in the history of this country, by raising a fund for a seamen's hospital. At this time (1897) the seafaring population of Cardiff (which is a quick despatch port, occupying also, perhaps, the foremost position in the world for export tonnage) averaged 6,000 daily. The new hospital had carried on its work unobtrusively, but most successfully, for thirty years. The new hospital was completed in the year 1905 at a cost of £28,399, in addition to £1,599 for furnishing, fitting-up, surgical instruments, etc. Of this sum £13,500 was received through the will of the most Honourable John Patrick Crichton Stuart, late Marquess of Bute, together with the greater part of the site. The new building contains three wards, with fifty-four beds in all. His Majesty the late King was graciously pleased to give his consent to the prefix "Royal" being used in the name of the new hospital.

Lord Merthyr, the President, besides having contributed to the building fund, permanently endowed a bed in the new hospital, and the committee of the hospital is happy in having a President who is always pleased to lend his support in spite of the many calls made on him in other directions.

